# Multifunctional Carboxymethyl Chitosan/Sodium Alginate/Fucoidan Hydrogel as a Dressing for Hemostasis and Skin Wound Healing

**DOI:** 10.3390/gels11120950

**Published:** 2025-11-26

**Authors:** Xinyue Gai, Yinghao Bi, Wen Zhao, Changlong Ren, Ming Chang, Miansong Zhang, Tingting Cui, Xue Liu, Airong Jia

**Affiliations:** 1 Biology Institute, Qilu University of Technology (Shandong Academy of Sciences), Jinan 250103, China; gaixinyue0329@163.com (X.G.); 10431221284@stu.qlu.edu.cn (Y.B.); 15005486183@163.com (W.Z.); rcl2863561955@163.com (C.R.); 17852647330@163.com (M.C.); zhangms@sdas.org (M.Z.); tingtingcui@sdas.org (T.C.); 2 Shandong Marine Functional Food Technology Innovation Center, Weihai 264305, China; 3 Fujian Ocean Innovation Center, Xiamen 361102, China

**Keywords:** hydrogel, carboxymethyl chitosan, sodium alginate, fucoidan, hemostasis, wound healing

## Abstract

Wound healing is a complicated process that involves hemostasis, antibacterial defense, and tissue regeneration. Conventional treatment methods, such as surgical suturing, have inherent limitations, necessitating the exploration of new ones. Hydrogels can create a moist environment that facilitates wound healing, making them an ideal material for wound healing. In this study, a procoagulant polysaccharide mixture (carboxymethyl chitosan/sodium alginate/fucoidan; CAF) was designed. Hydrogels were prepared using CAF and an oxidized starch/tannic acid blend (OT) at different ratios. Through comprehensive evaluations, such as gelation time, swelling capacity, and antibacterial efficacy, an optimal hydrogel (COT) was identified. This COT hydrogel was formed by mixing 3% CAF and OT solutions at a ratio of 2:1 (*v*/*v*). The associated gelation process occurred rapidly within 13 s. COT hydrogel exhibited self-healing properties, and a high swelling rate (~3109 ± 74%). It also demonstrated high antibacterial activity, facilitating enhanced protection against infection. Additionally, COT hydrogel exhibited biocompatibility and biosafety. COT hydrogel demonstrated low cytotoxicity on mice fibroblast cells (L929) and good hemocompatibility in vitro. Moreover, in vivo evaluations revealed that it did not cause skin irritation or allergic reactions. Importantly, COT hydrogel significantly outperformed the commercially available hydrogel with its hemostatic and wound healing performance (*p* < 0.001, *p* < 0.01).

## 1. Introduction

The skin covers the entire external surface of the human body and can be considered its largest organ, and it plays a key role in both homeostasis and the prevention of invasion by microorganisms [1]. However, certain traumatic events (for example, violent accidents/incidents, surgical operations, etc.) can result in severe skin injuries and (occasionally) full-thickness skin defects [2]. If not treated properly, major skin defects may develop into chronic wounds that are difficult to heal. This can lead to a range of serious health complications, including inflammation, secondary damage, and bacterial infection, and (in extreme cases) disability or death [3]. Additionally, uncontrollable bleeding caused by trauma is another major cause of death worldwide, and rapid hemostatic intervention improves the survival of injured patients [4,5]. To date, the most common method for treating skin wounds is surgical suturing [6]. However, suturing procedures are usually performed by trained professionals, and these specialized medical interventions are often delayed in critical environments like battlefields. Consequently, there is an imperative to explore new wound treatment methods, and wound dressings with novel properties have subsequently been developed. In recent years, multifunctional dressings with antibacterial, hemostatic, and wound-healing properties have become a significant research focus of the medical community.

Recent research has revealed that wounds are more likely to heal in humid environments [7]. Hence, wet wound dressings are gradually attracting more and more attention. Ideally, wound dressings should meet the following criteria: excellent biocompatibility without inducing inflammation or toxicity; superior hemostatic properties; effective inhibition of bacterial growth; and the ability to maintain a moist environment to support wound healing [8,9]. Hydrogels have been shown to exhibit outstanding swelling properties and biocompatibility, and they create a moist environment that facilitates wound healing. Hence, hydrogels are an ideal choice for wound dressings.

Marine polysaccharides are commonly utilized in hydrogels due to their excellent biocompatibility and biosafety [10,11,12]. For example, carboxymethyl chitosan (CMCS) was demonstrated to exhibit enhanced solubility, biocompatibility, biodegradability, hemostatic and antibacterial effects [13,14]. Sodium alginate (SA) also exhibited excellent biocompatibility, and it was subsequently shown to facilitate wound healing [12,15]. Fucoidan (Fuc) was observed to exhibit procoagulant activity at low concentrations, although it did also exhibit anticoagulant activity at high concentrations [16]. Moreover, Fuc can effectively regulate inflammation and accelerate tissue regeneration [17]. Because of their favorable properties, hydrogels based on polysaccharides have garnered significant attention in tissue engineering and medical applications. Xie et al. [14] reported the development of carboxymethyl chitosan/oxidized dextran/sodium alginate hydrogels that effectively prevented Staphylococcus aureus-induced wound infections. Intini et al. [18] designed a 3D-printed hydrogel dressing based on chitosan that exhibited excellent cytocompatibility and biocompatibility, and this dressing was observed to promote wound healing in clinical evaluations. Liang et al. [19] developed a multifunctional hyaluronic acid-based hydrogel dressing that exhibited antimicrobial and free radical scavenging properties. This innovative dressing enhanced the expression of CD31 growth factor, thereby promoting neovascularization in the wound area.

However, despite extensive research on hydrogels, several limitations remain to be addressed. The gelation time is currently too long, ranging from minutes to tens of minutes, which significantly compromises clinical operational efficiency [20]. The slow gelation process also limits the in situ shaping capability at wound sites, thereby hindering the ability to conform closely to complex or irregular wound geometries [21]. Additionally, the complex preparation process for hydrogels of interest often involves costly and toxic reagents, limiting the feasibility of industrial manufacturing [22,23]. More importantly, many of these products exhibit limited efficacy in promoting wound healing and achieving hemostasis [24]. Therefore, there is an urgent need to develop new, high-performance, efficacious hydrogels.

In the present study, we aimed to address these challenges through the design of novel hydrogels. First, 16 polysaccharide mixtures (comprising CMCS, SA, and Fuc) with different ratios were designed using an orthogonal array. Their procoagulant activity was evaluated, and the mixture with the shortest coagulation time (named CAF) was selected. Hydrogels were then prepared using CAF as the primary material, with oxidized starch (OS)/tannic acid (TA) blends (OT) serving as the cross-linker. OS acts as a cross-linking agent by forming imine bonds with the amino groups of CAF via a Schiff base reaction, while TA further reinforces the network through multi-site hydrogen bonding and hydrophobic interactions with the polysaccharide chains. Through comprehensive evaluations, such as gelation time, swelling capacity, and antibacterial efficacy, an optimal hydrogel named COT was developed and selected for further analysis. These additional studies systematically assessed its biocompatibility, biosafety, hemostatic effects, and wound healing performance, and the results were compared with commercially available hydrogel products (Hydrosorb Gel, Paul Hartmann Co., Ltd., Heidenheim, Germany). Through these systematic studies, we demonstrated that COT was a high-performance, safe, and effective hydrogel wound dressing.

## 2. Results and Discussion

### 2.1. Preparation of COT Hydrogels

#### 2.1.1. Preparation of Polysaccharide Mixture CAF

**Figure 1:**
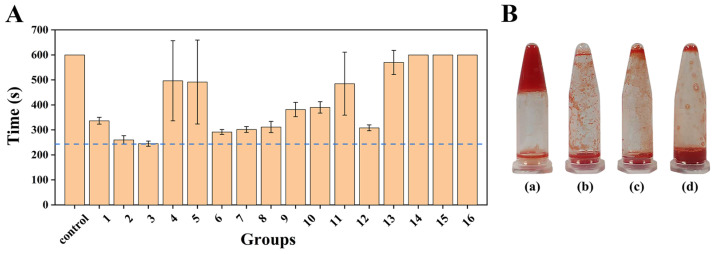
The procoagulant activity of the polysaccharide mixtures. (**A**) Clotting times of 16 polysaccharide mixtures (mean ± SD, *n* = 6). (**B**) Comparison of the coagulation effects of three hemostatic materials (*n* = 3): (a) CAF, (b) Yunnan Baiyao, (c) Chitosan hemostatic powder, (d) Deionized water.

Marine polysaccharides demonstrate great promise for wound dressing development. Among them, CMCS offers antibacterial and hemostatic properties [14], SA can form a moist gel matrix that promotes wound healing [12,15], and Fuc effectively regulates inflammation and accelerates tissue regeneration [17]. The combination of these three polysaccharides is expected to yield synergistic effects across multiple critical stages—including hemostasis, antibacterial action, and tissue regeneration, thereby constructing a wound dressing with performance surpassing that of a single component. Accordingly, 16 polysaccharide mixtures with varying mass ratios of CMCS, SA, and Fuc were designed. The procoagulant activity was evaluated to select the optimal ratio for the subsequent preparation of hydrogels. Figure 1A illustrated the clotting times of 16 polysaccharide mixtures, with mixture 3 (CMCS:SA:Fuc = 3:0.5:0.001 (*w*/*w*), named CAF) exhibiting the shortest time. CAF demonstrated remarkable clotting efficacy, whereas the commercially available hemostatic powders (Yunnan Baiyao and chitosan hemostatic powder) failed to achieve complete blood coagulation within the same time (Figure 1B). Consequently, CAF serves as an effective procoagulant polysaccharide mixture.

#### 2.1.2. Preparation of OT Cross-Linking Agent

**Figure 2:**
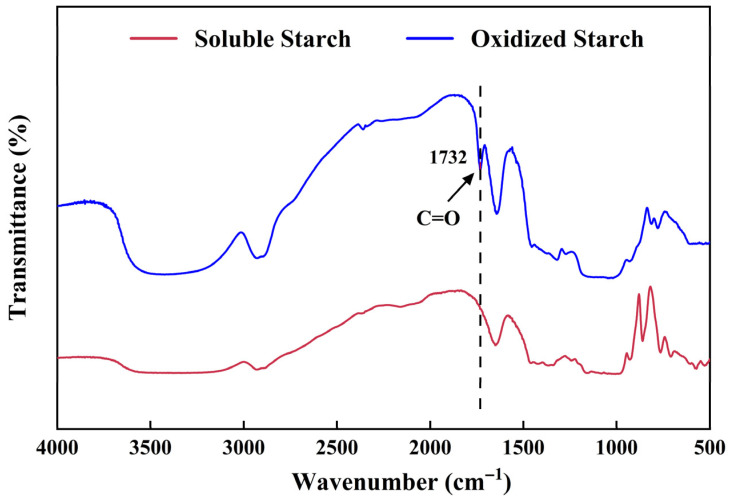
FTIR spectra of soluble starch and oxidized starch.

A qualified hydrogel dressing should possess a cross-linked structure to achieve adequate mechanical strength [14]. OS has the potential to act as an inter-crosslinking agent because its aldehyde groups can rapidly form imine bonds with the amino groups of CAF through a Schiff base reaction [14]. It can be derived from the oxidation of soluble starch. Figure 2 presented the Fourier-transform infrared (FTIR) spectra of soluble starch and OS. It is worth noting that the spectra were intentionally recorded with high signal intensity to accentuate the relatively weak carbonyl absorption. A clear peak at 1732 cm^−1^ was observed, assigned to the aldehyde stretching vibration [25], confirming the successful preparation of OS. The aldehyde group content was 76.97 ± 0.71%. TA has the potential to cross-link polysaccharide macromolecules at multiple binding sites via hydrogen bonding and hydrophobic interactions [26]. OT solution, consisting of 2% OS and 5% TA, was then prepared.

#### 2.1.3. Preparation of the Hydrogels

**Figure 3:**
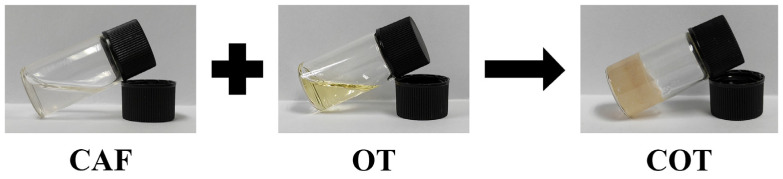
Schematic illustration of the preparation of COT hydrogels.

COT hydrogels were generated via the interactions between CAF and OT. The entire preparation process for COT hydrogels was illustrated in Figure 3.

### 2.2. Characterizations of COT Hydrogels

#### 2.2.1. Determination of the Gelation Time

In general, differences in hydrogel gelation times can be directly attributed to variations in polymer concentration and crosslinking ratios. In the present study, CAF concentration and the mixing ratio significantly influenced the gelation time of the resulting COT hydrogel. Specifically, hydrogel failed to form within 3 min at a 2% CAF concentration with mixing ratios of 1:2, 1:1, and 8:1 (COT-2-0.5, COT-2-1, COT-2-8; Table 1), and at a 3% CAF concentration with mixing ratios of 1:2 and 8:1 (COT-3-0.5, COT-3-8; Table 2). Under all other ratio conditions, the resulting hydrogel completed gelation within 80 s. Based on these findings, COT-2-3, COT-2-4, COT-3-2, and COT-3-3, which exhibited the shortest gelation times (COT-3-2 was especially short at 13 s), were selected for further experimentation. Notably, the rapid gelation times obtained were significantly shorter than those reported for other wound healing hydrogels, which typically range from 5 to 15 min [27,28]. In general, shorter gelation times are preferred for quicker application and improved wound conformability [29].

#### 2.2.2. Microstructure of COT Hydrogels

**Figure 4:**
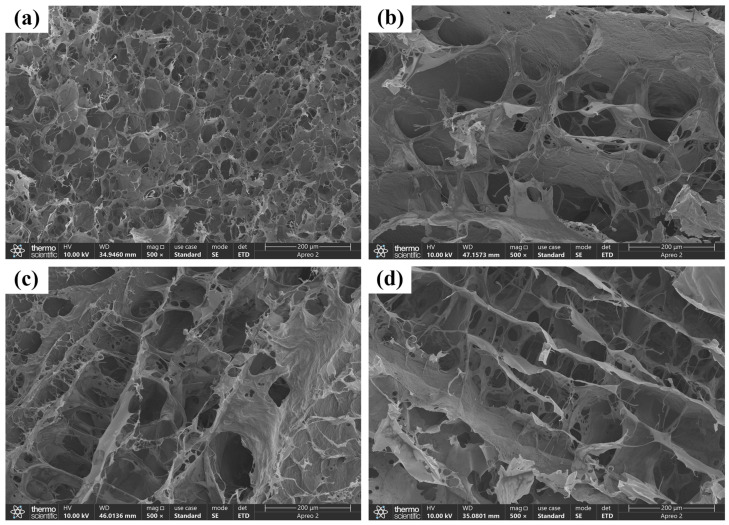
SEM of the COT hydrogels. (**a**) COT-2-3 hydrogel. (**b**) COT-2-4 hydrogel. (**c**) COT-3-2 hydrogel. (**d**) COT-3-3 hydrogel. Scale bar: 200 μm.

The porous network structure of hydrogels facilitates the absorption of body fluids and blood, and the adhesion of red blood cells and blood clot formation, thereby providing effective hemostasis. Scanning electron microscopy (SEM) was used to examine the hydrogel’s microscopic network structure in the freeze-dried state. Results indicated that the prepared hydrogels exhibited uniform pore structures. Specifically, the average pore diameters were as follows: COT-2-3, 58 ± 9.8 μm; COT-2-4, 100 ± 11.54 μm; COT-3-2, 120 ± 25.55 μm; and COT-3-3, 136 ± 18.88 μm (Figure 4). For biomedical materials utilized in wound healing applications, larger pore sizes can ensure adequate oxygen supply, thereby facilitating wound healing. However, excessively large pores may compromise the structural integrity and mechanical stability of the hydrogel, potentially affecting its overall functionality [30,31]. Hence, selection of an appropriate hydrogel composition is of paramount importance. As reported in the existing literature, the pore sizes of hydrogels typically range from 50 μm to 150 μm [28,32,33].

#### 2.2.3. FTIR and Rheological Analysis

**Figure 5:**
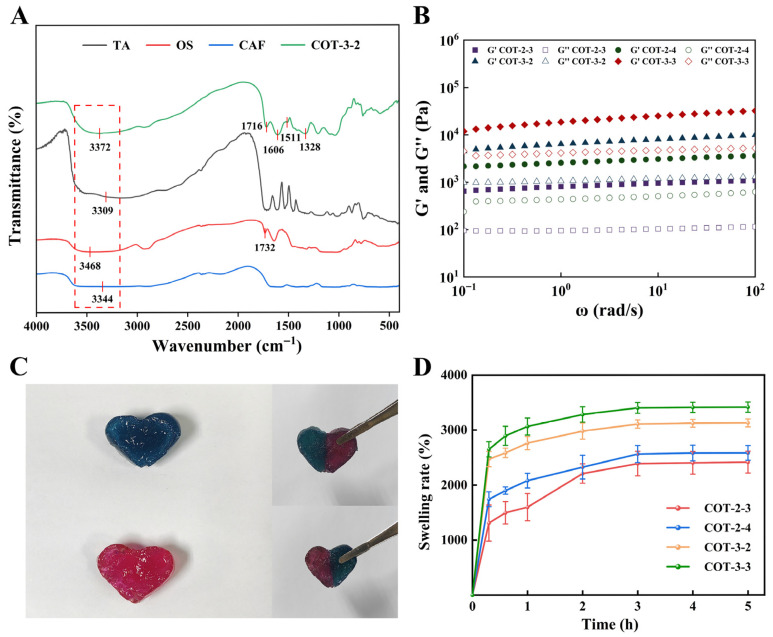
Characterizations of COT hydrogels. (**A**) FTIR spectra of CAF, OS, TA, and COT-3-2 hydrogel. (**B**) Frequency scanning of COT hydrogels. (**C**) The self-healing property of COT hydrogel (*n* = 3). (**D**) Swelling rate of the COT hydrogels (mean ± SD, *n* = 3).

FTIR spectra of CAF, OS, TA and the hydrogel (take COT-3-2 as an example) were shown in Figure 5A. A broad and intense peak observed in the range of 3600–3250 cm^−1^ was attributed to the stretching vibrations of O-H/N-H groups, indicating abundant hydrophilic groups and an extensive hydrogen bonding network [34]. The peak at 1718 cm^−1^ was attributed to the carbonyl (C=O) functional group [25]. In the hydrogel, the peak at 1607 cm^−1^ was attributed the imine bond (C=N) and also the amide I band. The decrease in the wavenumber might be caused by hydrogen bonding. Meanwhile, the peak near 1511 cm^−1^ was attributed to the amide II band [14]. Furthermore, the absorption peak at 1326 cm^−1^ was assigned to the C-N stretching vibration [35]. The hydrogen bonds, imine bonds and amide bonds confirm the crosslinking networks in the hydrogel.

To assess the viscoelastic characteristics of the hydrogels, frequency sweep tests were performed (Figure 5B). The results demonstrated that, throughout the entire frequency spectrum, the storage modulus (G′) of all hydrogels was significantly higher than the loss modulus (G″), indicating that these hydrogels predominantly exhibited elastic characteristics and maintained structural stability. Specifically, COT-3-3 exhibited higher G′ and G″ values (>10^4^ Pa), reflecting robust crosslinking density and mechanical performance, followed by COT-3-2. Conversely, COT-2-3 and COT-2-4 displayed lower moduli, which imparted greater softness and flexibility to these hydrogels.

#### 2.2.4. Self-Healing Behavior of COT Hydrogels

The self-healing properties of hydrogels improve the durability and effectiveness of the dressing during long-term use, helping to repair cracks caused by friction or damage [36]. As an example, COT-3-2 was used to illustrate the self-healing behavior of the hydrogels. Two COT-3-2 hydrogel samples, one staining with trypan blue and the other with rhodamine B, were each divided into two halves. Subsequently, the respective halves were aligned and incubated at room temperature without external stimuli. Upon gentle manipulation with tweezers, the recombined hydrogel was examined, confirming successful bonding at the gel interface and restoration of a structurally intact material. Moreover, the hydrogel did not separate under external force, confirming that COT-3-2 hydrogel exhibited significant self-healing behavior (Figure 5C).

#### 2.2.5. Swelling Behavior of COT Hydrogels

A hydrogel immersed in PBS will swell over time until it reaches swelling equilibrium, and this is a key characteristic [37]. The swelling ratio (obtained from before and after weight measurements) reflects the water content and water retention capacity of a hydrogel. Time-dependent swelling curves revealed that COT-3-2 and COT-3-3 hydrogels exhibited higher swelling ratios, reaching 3109 ± 74% and 3403 ± 99%, respectively. The swelling ratios of COT-2-3 and COT-2-4 hydrogels were lower in comparison, at 2389 ± 223% and 2563 ± 155%, respectively (Figure 5D). Valipour et al. [38] reported a swelling ratio of 740% to 1280% for their hydrogels, and Cai et al. [39] reported a swelling ratio of 800% to 3300% for their hydrogels. Hence, the COT hydrogel preparations reported here exhibited good swelling ratios. Because COT hydrogels exhibit superior water absorption and swelling properties, they should be able to maintain an optimal moist environment when used in wound dressings, helping to promote healing.

### 2.3. Antimicrobial Performance

**Figure 6:**
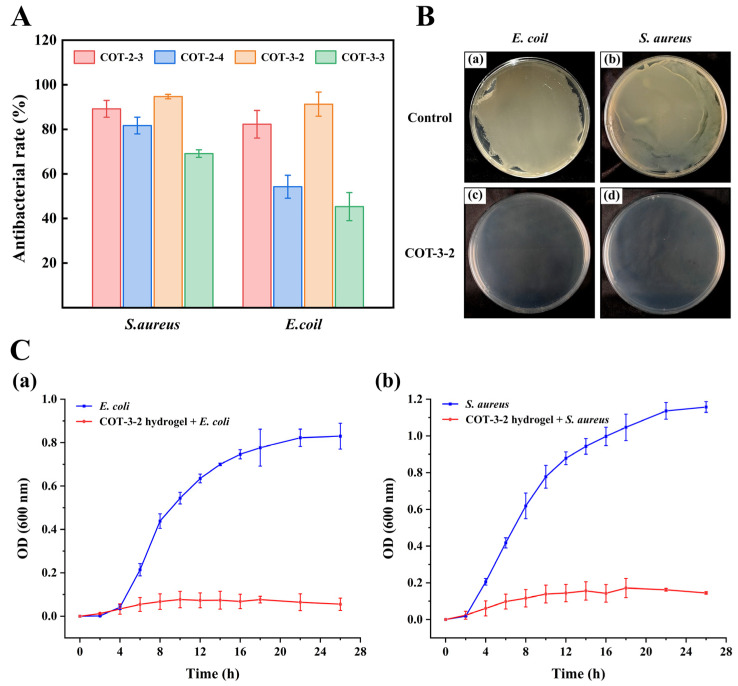
Antimicrobial properties of COT hydrogels. (**A**) The antibacteria rate of COT hydrogels on *E. coli* and *S. aureus* (mean ± SD, *n* = 6). (**B**) Photographs of survival bacteria clones on agar plates after contacting with COT-3-2 (*n* = 3): (a) *E. coli*, (b) *S. aureus*, (c) COT-3-2 hydrogel + *E. coli*, (d) COT-3-2 hydrogel + *S. aureus*. (**C**) Effect of COT-3-2 hydrogel on the growth of *E. coli* (a) and *S. aureus* (b) (mean ± SD, *n* = 6).

Wound dressings with antibacterial properties can significantly reduce bacterial contamination, and thereby mitigate inflammatory responses and fostering a cleaner and more conducive microenvironment for wound healing [40]. As shown in Figure 6A, COT-3-2 hydrogel exhibited good antibacterial efficacy against *E*. *coli* and *S*. *aureus*, two prevalent clinical pathogens that frequently cause wound infections. Specifically, the inhibitory rates of COT-3-2 against *E*. *coli* and *S*. *aureus* were 91.30 ± 5.39% and 94.71 ± 0.99%, respectively. After contacting COT-3-2, almost no surviving bacterial colonies were observed on the agar plates (Figure 6B). To further verify the antibacterial effect of the hydrogel, the effects of COT-3-2 on the growth curve analysis of *E. coli* and *S. aureus* were investigated (Figure 6C). The OD_600_ of the control group increased rapidly, following the typical bacterial growth curve. In contrast, the growth of both *E. coli* and *S. aureus* was significantly inhibited by the COT-3-2 hydrogel, as evidenced by a minimal increase in OD_600_ values and a rapid entry into a stationary phase.

The antibacterial activity of the hydrogel may be attributed to several key factors. First, the cationic amino groups carried by CMCS can adsorb negatively charged bacteria by electrostatic force and accumulate on the bacterial cell wall, thus inhibiting the growth of bacteria [8]. Second, TA, which contains a large number of phenolic hydroxyl groups, has been proved to inhibit the synthesis of the bacterial cell wall, showing strong antibacterial activity [8]. Additionally, the Schiff base formed by the amino groups and aldehyde groups has also been reported to exhibit good antibacterial activity [41]. The multifunctional hydrogel composed of CMCS, SA, and oxidized dextran developed by Xie et al. [14] exhibited inhibition rates of 26.67% against *E. coli* and 29.14% against *S. aureus*. Similarly, the CS/AgCl hydrogel prepared by Taghizadeh et al. exhibited inhibition rates below 50% for both these bacterial strains [42]. Thus, COT-3-2 hydrogel exhibited good antibacterial activity.

Considering all the indicators discussed above, COT-3-2 hydrogel exhibited better characteristics in terms of gelation time, microscopic morphology, self-healing capability, swelling behavior, and antibacterial efficacy. Consequently, COT-3-2 hydrogel (hereafter designated as ‘COT hydrogel’) was selected for further experiments.

### 2.4. In Vitro Biocompatibility

#### 2.4.1. In Vitro Hemocompatibility

**Figure 7:**
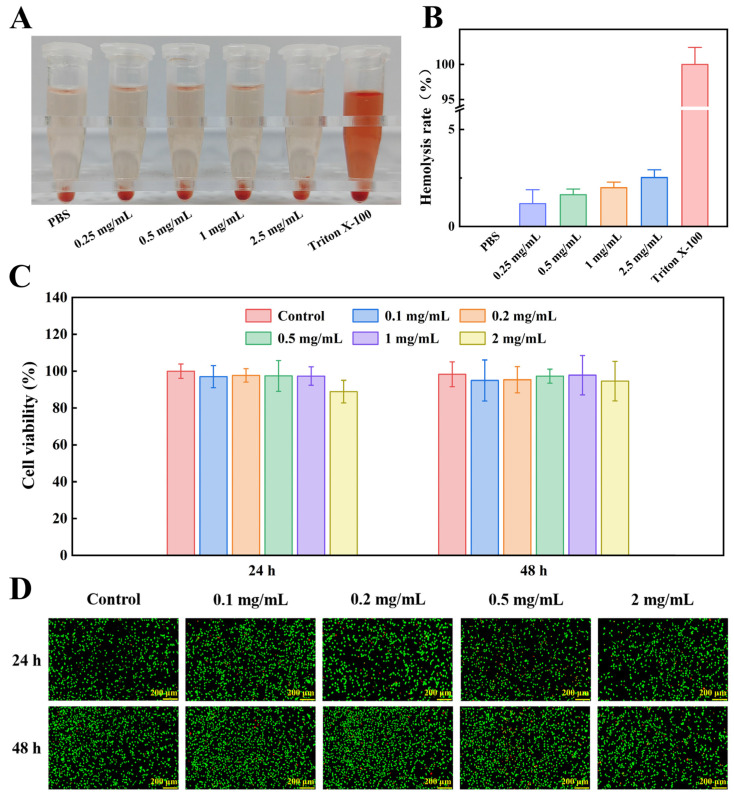
Biocompatibility of COT hydrogel. (**A**) Photographs of erythrocytes after different treatments in hemolysis analysis. (**B**) Hemolysis rate of COT hydrogel (mean ± SD, *n* = 6). (**C**) Cell viability of L929 cells cultured with the extracts of COT hydrogel (mean ± SD, *n* = 6). (**D**) Live/Dead staining of L929 cells treated with the extracts of COT hydrogel.

To be utilized in the biomedical field, biomaterials must exhibit excellent biocompatibility [43]. To evaluate hemocompatibility in vitro, hemolysis analysis was used to assess the interaction between hydrogel and erythrocytes. As a positive control of hemolytic activity, 0.1% Triton X-100 was also tested (with PBS used as a negative control). After testing, the supernatant color remained clear and transparent, indicating no hemolysis (consistent with the negative control group). In contrast, the positive control group presented a red color, indicating hemolysis (Figure 7A). The resulting hemolysis rates are shown in Figure 7B. The observed hemolysis rates of all hydrogel groups were below 5%, meeting the international standards for biomaterials [44]. Thus, COT hydrogel exhibited hemocompatibility for applications as a hemostatic dressing.

#### 2.4.2. In Vitro Cytocompatibility

Cell survival and growth are critical for the regeneration of skin tissue during wound healing, necessitating that hydrogels exhibit excellent biocompatibility [45]. Hydrogel cytocompatibility was evaluated by assessing cell viability in the hydrogel leachate (Figure 7C). After co-incubation with L929 cells for 24 h and 48 h, the cell survival rate in the group treated with hydrogel leachate was slightly reduced (compared to the control group). The overall > 85% survival rate observed indicated a relatively high level of biocompatibility.

To further evaluate the biocompatibility of the hydrogel, cell morphology was assessed using a live/dead cell staining method (green fluorescence, viable cells; red fluorescence, apoptotic cells). After co-culturing with hydrogel leachate for 24 h and 48 h (Figure 7D), only a small number of red nuclei were observed in the hydrogel leachate-treated groups (compared to the control group), which is consistent with the findings from the CCK-8 assay. Together, the above results provide evidence that COT hydrogel exhibits good biocompatibility and may support skin tissue regeneration during wound healing.

### 2.5. Skin Irritation Test

**Figure 8:**
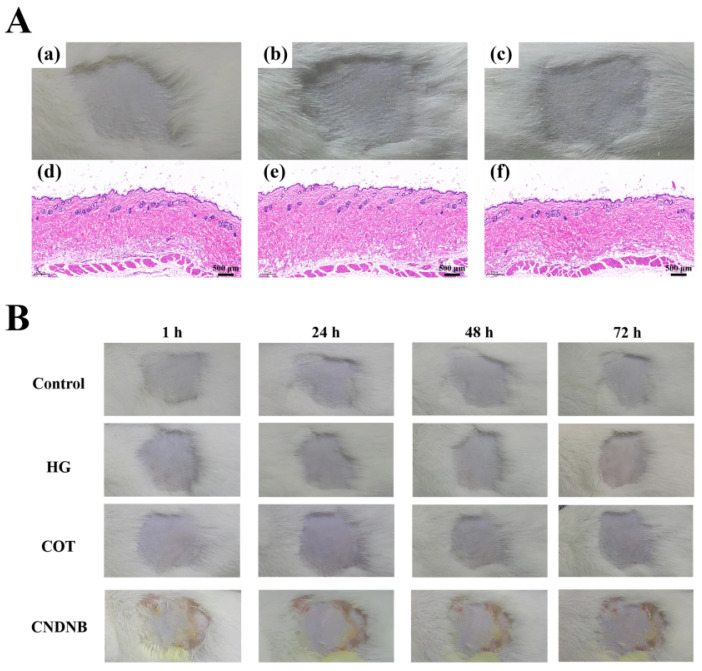
Mouse skin evaluation for irritation and allergenicity response. (**A**) Skin irritation test (after 72 h, *n* = 10): (a) The control group. (b) The HG group. (c) The COT hydrogel group. (d–f) H&E staining of skin tissues from the control, the HG and the COT groups in the irritant tests (H&E, ×10 magnification). (**B**) Skin allergenicity test (*n* = 10).

To evaluate the safety profile of the COT hydrogel, a skin irritation test was conducted on intact mouse skin. The mice were randomly divided into three groups: the control group (treated with deionized water), the HG group (treated with a commercially available hydrogel, Hydrosorb Gel (HG), for comparison), and the COT hydrogel group. Over the 14-day experimental period, the PII scores for the control and COT groups were consistently 0, indicating a negligible irritating effect. In the HG group, mice exhibited transient mild erythema was observed (approximately 1 h after treatment) from the 3rd to the 9th day. However, this symptom was fully resolved by the 10th day. The resulting PII scores obtained ranged from 0.3 to 1.0 (Table 3), indicating a negligible irritating or slightly irritating effect. During the 72 h observation period following the final application, no visible signs of irritation, including edema or erythema, were observed in any of the three groups. All PII scores remained at 0, confirming the negligible irritating effects (Table 3). Histopathological examination revealed that the skin structure in all groups remained intact and well-defined, with no atrophy or thinning of the spinous layer and stratum corneum, nor any pathological changes such as epidermal necrosis or inflammatory cell infiltration (Figure 8A). Hence, COT hydrogel elicited no skin irritation and outperformed the HG group, which elicited a slight irritation effect during the initial stage of use.

### 2.6. Skin Allergenicity Test

To further evaluate the safety profile of COT hydrogel, a skin allergenicity test was conducted on intact mouse skin. While obvious skin changes were observed in the group treated with CNDNB (a potent sensitizer that effectively induces skin inflammation), no changes were observed in the areas exposed to test formulations in any other group (Figure 8B). In all cases, the distinction between stimulated skin (obtained using CNDNB) and unaffected skin was clear. While the CNDNB group exhibited erythema (score, 1.5; indicative of a mild irritation response), no erythema (or other sign of irritation) was observed at the test sites treated with COT hydrogel, HG, or in the control group, all of which received a score of 0 (Table 4). Based on these findings, COT hydrogel and HG did not induce a skin allergy in mice. These findings demonstrate that COT hydrogel exhibited good skin compatibility, supporting its potential for use in therapeutic applications.

### 2.7. Hemostatic Evaluation

Hemostasis is the critical first step in wound healing, and it plays a crucial role in preventing complications (and promoting future recovery). Hemorrhagic shock is a leading cause of death, particularly in traffic accidents and combat-related injuries. Rapid hemostasis is essential for minimizing blood loss, preventing shock, and increasing survival rates.

The clotting time and blood clotting index (BCI) assays were initially used to evaluate the hemostatic performance of COT hydrogel (Table 5). The results showed that COT hydrogel significantly shortened the clotting time (*p* < 0.001) and achieved a low BCI of 11.52 ± 2.84%. Its coagulation efficacy was comparable to that of HG group, confirming its hemostatic potential.

**Figure 9:**
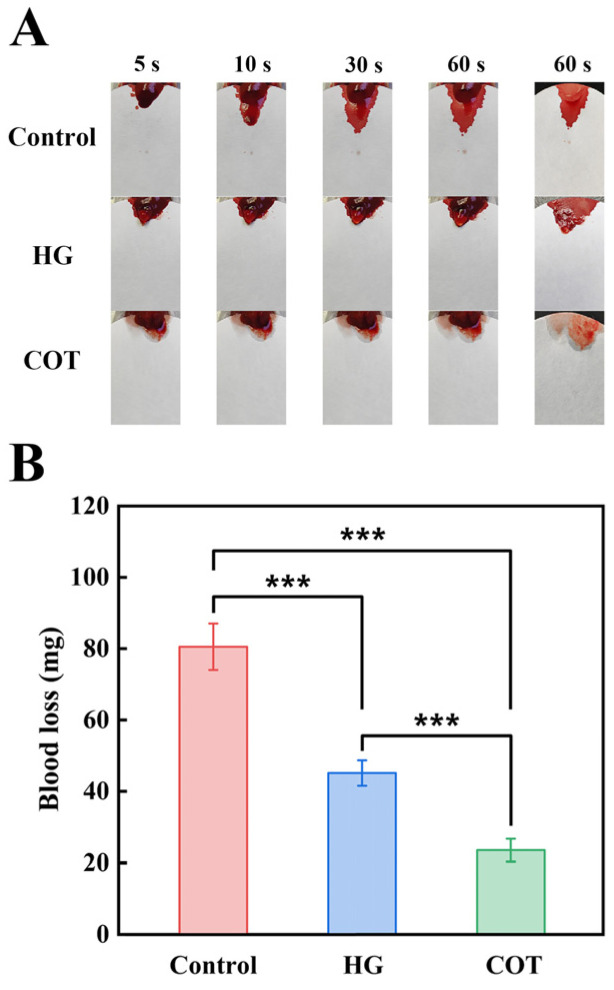
Analysis of hemostasis performance in vivo. (**A**) Representative images of the liver bleeding model and hemostasis. (**B**) Blood loss of liver injury model after different treatments (mean ± SD, *n* = 6). Significance: *** *p* < 0.001.

The hemostatic efficacy of COT hydrogel was further confirmed in a liver injury mouse model, which is a rigorous and widely accepted assessment of rapid hemostatic capability for skin wound dressings. As expected, blood loss was most serious in the control group (in which the livers of the mice were untreated after puncture). In comparison, mice in the HG and COT groups all showed rapid hemostatic effects (Figure 9A), with the COT hydrogel achieving nearly immediate hemostasis and minimal blood loss upon application. The observed reduction in bleeding volume (in comparison with the control group) was significant for both the HG group and the COT group (*p* < 0.001). Moreover, the COT group demonstrated a significantly lower bleeding volume compared to the HG group (*p* < 0.001) (Figure 9B). These findings indicate that COT hydrogel rapidly and effectively restricted continuous blood flow from the wound area, demonstrating better hemostatic performance compared to commercially available hydrogel HG.

### 2.8. Evaluation of Wound Healing In Vivo

**Figure 10:**
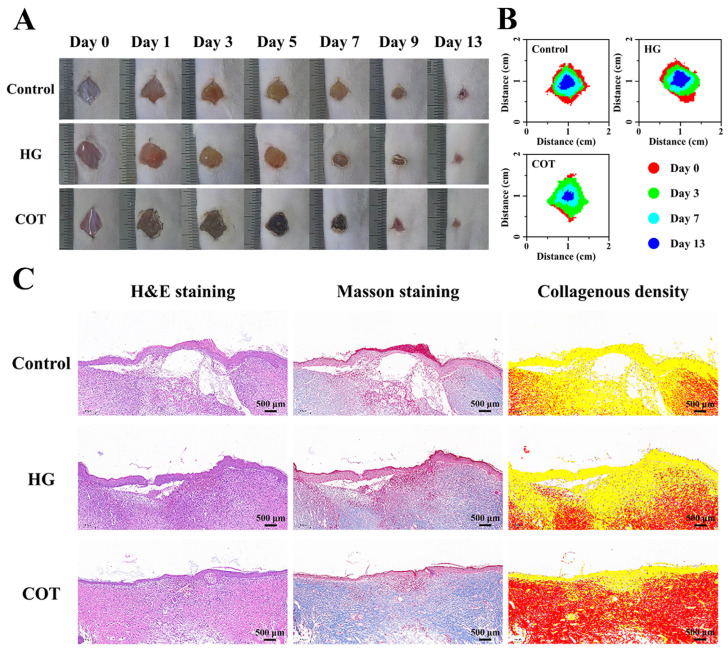
Evaluation and histological analysis of wound healing in vivo (*n* = 6). (**A**) Representative photographs of wounds with different treatments. (**B**) Stacked charts of wounds with different treatments. (**C**) Histological observation of skin wounds after 13 days of treatment.

A cutaneous wound model was utilized to evaluate the wound repair efficacy of COT hydrogel. The wound sizes in the control (mice received no treatment), HG, and COT hydrogel groups were monitored over time to assess differences in wound closure rates (Figure 10A). By day 13, the wound closure rate of the COT group was 92.69 ± 2.61%. This was significantly higher than the closure rate obtained in the control group (80.49 ± 4.02%; *p* < 0.01) or that obtained in the HG group (82.11 ± 3.36%; *p* < 0.01) (Figure 10B). A more comprehensive assessment of the healing process was provided by histological analysis. H&E and Masson staining of wound tissue sections revealed significant dermal and epidermal regeneration in the COT group after 13 days of healing (Figure 10C). Conversely, regeneration was less pronounced in both the control and HG groups. Protein deposition is an important indicator of tissue remodeling [46]. Masson staining was used to investigate differences in protein deposition between the treatment groups during wound healing. While sparse in control and HG groups, protein deposition was abundant in the COT group (Figure 10C). These findings provide evidence that COT hydrogel effectively promoted wound healing and skin regeneration, and that its performance significantly surpasses that of HG, a commercially available hydrogel.

## 3. Conclusions

As the most extensive and exposed organ, the skin acts as a physical barrier, maintaining homeostasis and preventing pathogen invasion [1]. Skin wounds can lead to numerous health complications, including inflammation and bacterial infection. If left untreated, these wounds can develop into chronic wounds that are difficult to heal. Additionally, the uncontrollable bleeding that accompanies a skin trauma is a major cause of death worldwide, and rapid hemostatic intervention can improve survival of the injured patients [4,5]. Therefore, the development of bioactive dressings with hemostatic properties, antibacterial capabilities, and wound healing activities has emerged as a significant research focus within the medical community.

In this paper, a high-performance hydrogel wound dressing (COT hydrogel) was prepared and evaluated. COT hydrogel exhibited a rapid gelation process (within 13 s) and good self-healing and swelling properties. Moreover, the inhibition rates of COT hydrogel against *E. coli* and *S. aureus* exceeded 90%, effectively protecting wounds from pathogenic bacterial infections. The hemolysis rate of the COT hydrogel was also less than 5%, thereby meeting international biomaterial standards and demonstrating hemocompatibility. Cytocompatibility tests indicated that COT hydrogel exhibits low cytotoxicity on mice fibroblast cells (L929). Additionally, in vivo evaluations confirmed that COT hydrogel did not cause skin irritation and allergic reactions. Thus, COT hydrogel exhibits good biocompatibility and biosafety. Importantly, the hemostatic effect and wound healing performance of COT hydrogel were significantly better than those of a commercially available hydrogel (*p* < 0.001, *p* < 0.01, respectively). In summary, COT hydrogel is a safe, effective, and high-performance wound dressing with promising application prospects in the field of wound healing.

## 4. Materials and Methods

### 4.1. Materials

CMCS (arboxylation ≥ 80%; the average weight molecular mass, ~18 kDa), Tannic acid (TA) and 1-chloro-2, 4-dinitrobenzene (CNDNB) were purchased from Aladdin Reagent Co., Ltd. (Shanghai, China). SA (98%; the ratio of mannuronic and guluronic units (M/G), ~1.56; ~200 kDa) and soluble starch (solubility, 500 mg/mL in H_2_O; ~15 kDa) were obtained from Yuanye Bio-Technology Co., Ltd. (Shanghai, China). Fuc (fucose, 71.89 (mol%); Sulfate content, 28.24%; ~90 kDa) was from Rensheng Pharmaceutical Group Co. Ltd. (Weihai, China). Yunnan Baiyao was obtained from Yunnan Baiyao Group Co., Ltd. (Kunming, China). Chitosan hemostatic powder was from Qingdao biotemed Biomaterials Co., Ltd. (Qingdao, China). Sodium periodate was from Macklin Biochemical Co., Ltd. (Shanghai, China). Hydrosorb Gel (HG) was from Paul Hartmann Co., Ltd. (Heidenheim, Germany).

Rabbit blood and rabbit red blood cells (4%) were obtained from SenBeiJia Biological Technology Co., Ltd. (Nanjing, China). Dulbecco’s Modified Eagle’s Medium (DMEM) was obtained from Procell Life Science & Technology Co., Ltd. (Wuhan, China). The Live/Dead cell dichromatic stain was from Elabscience Biotechnology Co., Ltd. (Wuhan, China). The Cell counting kit-8 (CCK-8) was purchased from Ding Guo Chang Sheng Biotechnology Co., Ltd. (Beijing, China). Mice fibroblast cells (L929) were obtained from the Institute of Biochemistry and Cell Biology (Shanghai, China).

Kunming (KM) mice (4–5 weeks) were acquired from Pengyue Experimental Animal Breeding Co., Ltd. (Jinan, China).

### 4.2. Preperation of COT Hydrogels

#### 4.2.1. Preparation of Polysaccharide Mixture CAF

CMCS, SA and Fuc were used as raw materials, and each was set at four different concentration levels (Table 6). Polysaccharide mixtures with different ratios were designed using an orthogonal array L_16_(4^5^) (Table 7). Specifically, CMCS, SA and Fuc were first dissolved at the corresponding concentrations, mixed in equal volumes, and then thoroughly stirred to ensure homogeneity. Thereafter, the resultant mixtures were freeze-dried and 16 polysaccharide mixtures were obtained.

The procoagulant activity of polysaccharide mixtures was evaluated by measuring coagulation time. 100 μL of sodium citrate-anticoagulated rabbit blood was mixed with 100 μL polysaccharide mixture (10 mg/mL) and 100 μL CaCl_2_ solution (37 °C, 0.2 mol/L). The tube was tilted every 10 s to observe the fluidity, and timing was stopped upon complete coagulation. In the control group, the polysaccharide mixture was replaced with deionized water. The polysaccharide mixture with the shortest coagulation time was selected and compared with the commercially available hemostatic powder (Yunnan Baiyao and chitosan hemostatic powder).

#### 4.2.2. Preparation of Oxidized Starch and OT Cross-Linking Agent

OS was prepared following the method by Zhang et al. [47] with minor modifications [38]. Briefly, soluble starch (10 g) was dissolved in deionized water, and 8.5 g NaIO_4_ was then added. The resulting reaction was allowed to proceed at room temperature (avoiding direct light) for 4 h, and subsequently terminated by the addition of 1 mL ethylene glycol for another 30 min. The reaction solution was then dialyzed (molecular weight cutoff, 7000 Da) and lyophilized to obtain OS.

Next, the aldehyde group content of OS was determined using the rapid quantitative alkali consumption method [48]. Dried OS (0.15 g) was suspended in 10 mL of 0.25 M NaOH solution in an Erlenmeyer flask. The flask was then swirled in a water bath at 70 °C for 2 min and rapidly cooled under running tap water for 1 min (with continuous swirling). Subsequently, 15 mL of 0.125 M H_2_SO_4_, 30 mL of deionized water, and 1 mL of 0.2% phenolphthalein indicator were added sequentially. Finally, the resulting acidic solution was titrated using 0.25 M NaOH solution. The percentage of dialdehyde units was calculated as follows:

(1) Da (%)=C1V1−2C2V2W161×1000×100%

where C_1_ (M) and C_2_ (M) represent the normality of NaOH and H_2_SO_4_, respectively. V_1_ (mL) and V_2_ (mL) represent the total volume of NaOH and H_2_SO_4_, respectively. W (g) is the dry weight of the OS sample, and 161 is the average molecular weight of the repeat unit in OS. The experiments were performed in triplicate.

The FTIR spectra of soluble starch and OS were measured with a Nicolet Nexus 470 infrared spectrometer (Madison, WI, USA) in the scanning range of 400~4000 cm^−1^ [38].

A cross-linking agent OT solution, consisting of 2% OS and 5% TA (*w*/*v*), was then prepared.

#### 4.2.3. Preparation of the Hydrogels

To prepare COT hydrogels for our preliminary investigations, 2% and 3% CAF solutions were thoroughly mixed with OT solution at ratios of 1:2, 1:1, 2:1, 3:1, 4:1, and 8:1 (*v*/*v*). The resulting mixtures were designated as COT-2-n and COT-3-n, respectively.

### 4.3. Characterization of COT Hydrogels

#### 4.3.1. Determination of Gelation Time

The inverted test tube method was used to record the gelation time [8]. Gelation times exceeding 3 min were not recorded.

#### 4.3.2. SEM Analysis

The microscopic network structures of the hydrogels were examined using SEM. First, the freeze-dried hydrogel samples were cut into thin sections. These sections were subsequently affixed to the sample holder using conductive adhesive and then sputter-coated with gold. All SEM images were acquired at an operating voltage of 5 kV.

#### 4.3.3. FTIR Spectroscopy Analysis

The FTIR spectra of hydrogels were measured as mentioned above.

#### 4.3.4. Rheological Properties Assay

Rheological properties of the hydrogels were characterized using a rheometer by monitoring the loss modulus (G″) and storage modulus (G′). All measurements were carried out at 37 °C using 20 mm parallel plates with a gap size of 1 mm. A volume of 0.3 mL of the hydrogel precursor solution was carefully dispensed between the parallel plates. Subsequently, frequency sweep analysis was conducted over a shear strain range of 1–500% and a shear frequency range of 0.1–100 rad/s.

#### 4.3.5. Self-Healing Properties of COT Hydrogels

The self-healing properties of COT hydrogels were evaluated by staining two hydrogel sections and then observing their reconnection [6]. The first heart-shaped hydrogel sample was stained with trypan blue, and the second heart-shaped hydrogel sample was stained with rhodamine B. Each hydrogel sample was then evenly divided into two halves. Finally, the differently stained halves were brought into contact and the self-healing characteristics of COT hydrogels were assessed through observations of the gel state.

#### 4.3.6. Swelling Analysis

Freeze-dried COT hydrogels were weighed and subsequently immersed in PBS (pH, 7.2) at room temperature [14]. At hourly intervals, the hydrogels were removed from the PBS solution, and excess surface moisture was carefully blotted using filter paper. The weight of the hydrogels was then recorded until a constant weight was achieved. The swelling rate was calculated as follows:

(2) $$Swelling\;rate\;(\%)=\frac{\left(W_{t}-W_{0}\right)}{W_{0}}\times 100\%$$

W_0_ and W_t_ represent the weights of initial and swelled hydrogels, respectively.

### 4.4. In Vitro Antibacterial Evaluation

The antibacterial activities of COT hydrogels were evaluated using *Escherichia coli* (*E. coli*) and *Staphylococcus aureus* (*S. aureus*) as representative species [14,49]. These bacterial strains were first cultured in Luria–Bertani (LB) liquid medium at 37 °C for 12 h. Next, 100 μL of COT hydrogel was dispensed into each well of a 48-well plate and allowed to fully solidify. The solidified hydrogels were then irradiated under ultraviolet (UV) light for 1 h to ensure complete sterilization. Next, 100 μL of bacterial suspension was added to each well. These samples were then incubated for an additional 12 h. As a control, we also incubated 100 μL of bacterial suspension without hydrogel. After 12 h, 900 μL of LB liquid medium was added to each well, and the cultures were further incubated for another 12 h. Finally, the optical density (OD) at 600 nm was measured for each well using a microplate reader (MK3, Thermo Scientific, Waltham, MA, USA). All antibacterial rates were calculated as follows:

(3) $$Antibacterial\;rate\;(\%)=\frac{\left(A_{c}-A_{s}\right)}{A_{c}}\times 100\%$$

where A_c_ and A_s_ represent the absorbances of the control group and the experimental group, respectively.

An additional batch was processed using COT-3-2 hydrogel following the same procedure. After the addition of 900 μL of LB medium, the surviving bacteria were re-suspended. 100 μL of the bacterial suspension was then plated onto an LB agar plate and incubated for 12 h, after which colony growth was observed.

The antibacterial activity of the hydrogel was further reflected by the bacterial growth curve [50]. The hydrogel COT-3-2 was irradiated under UV light for 1 h to ensure complete sterilization and subsequently incubated in 10 mL LB medium for 12 h to prepare the leaching solution. *E. coli* and *S. aureus* were then inoculated into the leaching solution. Finally, the cultured broths were incubated at 37 °C and shaken at 200 rpm in a rotary shaker (MB-T610, Zhejiang Meibi Instrument Co., Ltd., Jiaxing, China). The turbidity of the culture was monitored by measuring the OD_600_ at 2 h, 4 h, 6 h, 8 h, 10 h, 12 h, 14 h, 16 h, 18 h, 22 h, and 26 h.

### 4.5. Biocompatibility Performance Analyses

#### 4.5.1. In Vitro Hemolysis Analysis

The blood compatibility of the optimal COT hydrogel was evaluated using a hemolysis test [8,43]. For the experiments, 1.2 mL of PBS, 300 μL of a 4% rabbit red blood cell suspension, and different masses of freeze-dried COT hydrogel were added into 2 mL test tubes (COT hydrogel was tested at final concentrations of 0.25, 0.5, 1, and 2.5 mg/mL). All samples were incubated at 37 °C for 2 h, and then centrifuged at 10,000 rpm for 15 min. Finally, OD_540nm_ readings were measured using a microplate reader (MK3, Thermo Scientific, Waltham, MA, USA). PBS and 0.1% Triton X-100 (which exhibits a pronounced hemolytic effect; Habibi et al. [51] served as negative and positive controls, respectively. All hemolysis rates were calculated as follows:

(4) $$\mathrm{Hemolysis}\;\mathrm{rate}\;(\%)=\frac{\left(A_{s}-A_{b}\right)}{\left(A_{t}-A_{b}\right)}\times 100\%$$

where A_s_, A_b_, and A_t_ represented the absorbance of the COT hydrogel, the negative control, and the positive control, respectively.

#### 4.5.2. In Vitro Cell Compatibility

First, the hydrogel was irradiated under UV light for 1 h to ensure complete sterilization. The sterilized COT hydrogel was then incubated in DMEM for 24 h to prepare the leaching solutions. Next, L929 cells were seeded in a 96-well plate at a density of 5000 cells per well. After an initial incubation period of 24 h, the medium in each well was replaced with hydrogel leaching solution. After co-incubation periods of 24 h and 48 h, the leaching solution was refreshed in each well. To assess cell viability, CCK-8 solution (10 wt%) was added to each well, and the plates were incubated for a further 1.5 h. The OD_450nm_ was then measured using a microplate reader (MK3, Thermo Scientific). Cell viability was also assessed using a calcein-AM/PI double staining kit. The initial experimental procedure was as described above (for CCK-8). Next, L929 cells were incubated with calcein-AM (0.1 wt%) and PI (0.1 wt%) for an additional 30 min. Finally, the stained cells were observed and photographed using a fluorescence microscope (Nikon TS 100, Nikon, Tokyo, Japan).

### 4.6. Skin Irritation Analysis

The skin irritation potential of COT hydrogel was evaluated following the method of Sallam et al. [52] with minor modifications. A commercially available hydrogel (HG) was used for comparison. Male Kunming mice were housed in a controlled environment (23 ± 2 °C, 12/12 h light/dark cycles) with ad libitum access to food and water. After one week of acclimation, the mice were randomly divided into three groups: a control group; the HG group; and the COT hydrogel group. The dorsal flank area (2 × 2 cm^2^) of each mouse was shaved 24 h prior to the start of testing. Next, individual hydrogel samples were applied to the hair-free skin, covering an area of 4 cm^2^. In the control group, all mice were similarly treated with deionized water. The above application procedures were continued daily for 14 consecutive days. At 1 h post-application each day, skin assessments for erythema and edema were performed. Additionally, skin assessments for erythema and edema were performed at 24 h, 48 h, and 72 h following the final application. All skin reactions were assessed according to Table 8 and Table 9, and the degree of skin irritation in each mouse was quantified by calculating the Primary Skin Irritation Index (PII) as follows:

(5) PII=Primary dermal irritation score (aggregation score of each points)Total time points

At the end of the 72 h observation period, skin and subcutaneous tissue at the administration site of each mouse were excised for fixation, dehydration, clearing, and paraffin embedding. The tissue sections were subsequently stained with hematoxylin and eosin (H&E) and then examined for histopathological changes.

### 4.7. Skin Allergenicity Analysis

To evaluate the potential allergenic and sensitizing effects of COT hydrogel, skin sensitivity tests were performed [53]. First, mice were randomly divided into four groups: a control group (treated with deionized water); the HG group; the COT hydrogel group; and the CNDNB group (treated with 1% CNDNB, a potent sensitizer that effectively induces skin inflammatory responses). At 24 h before the start of testing, a 2 × 2 cm^2^ area was shaved on the back (flank) of each mouse. The experiment consisted of two phases: an induction phase and a challenge phase. During the induction phase, the appropriate test substance was applied to the shaved area of each mouse on days 0, 7, and 14. After 6 h, the test substances were removed, and the application sites were cleaned with warm water. In the challenge phase (on day 21), the appropriate test substance was applied to the shaved area on the opposite side of each mouse to induce a contact response. After 6 h, the area was rinsed with warm water. The tested skin patches were then observed at 1 h, 24 h, 48 h, and 72 h post-application to assess the occurrence of allergic reactions (in each individual mouse). In each case, skin reactions were assessed and categorized based on the Magnusson and Kligman scales (Table 10). Finally, allergenicity observation scores and sensitization rates were calculated.

### 4.8. Hemostatic Evaluation

The hemostatic efficacy of COT hydrogel was first evaluated by measuring the clotting time and BCI. The coagulation time was determined according to the procedure described in Section 4.2.1 “Preparation of Polysaccharide Mixture CAF” with minor modifications. Briefly, 500 μL COT or HG hydrogel was added into 100 μL of sodium citrate-anticoagulated rabbit blood, followed by the addition of 100 μL CaCl_2_ solution (37 °C, 0.02 mol/L). In the control group, an equal volume of blood was mixed directly with CaCl_2_ solution. The coagulation time was recorded upon clot formation.

BCI was performed according to the following procedure with minor modifications [54]: the COT or HG hydrogel (1 cm^2^) was pre-incubated at 37 °C for 5 min. 100 μL of sodium citrate-anticoagulated rabbit blood was dropped onto each sample, followed by the addition of 10 μL of CaCl_2_ solution (0.2 mol/L) at the same position. After incubating at 37 °C for 5 min, 10 mL of deionized water was added and incubated at 37 °C for another 10 min. The hemoglobin absorbance of non-adherent blood clots was measured at 542 nm (denoted as Abssample). The negative reference was sodium citrate-anticoagulated rabbit blood in deionized water (denoted as Absnegative). The BCI was calculated using this formula:

(6) $$BCI\;(\%)=\frac{Abssample}{Absnegative}\times 100\%$$

The hemostatic efficacy of COT hydrogel was further evaluated using a mouse liver injury model. First, the mice were randomly divided into three groups: the blank control group; the HG group; and the COT group. To generate the liver injury model, the upper abdomen of each mouse was surgically opened to expose the liver, and a 1 cm incision was made in the liver tissue. Subsequently, 1 mL of either COT hydrogel or HG was immediately applied to the wound site (the control group received no treatment). Immediately following completion of the procedure, the bleeding status was recorded at specified time points (5 s, 10 s, 30 s, and 60 s), and the final blood loss at 60 s was evaluated. Specifically, a pre-weighed filter paper was positioned under the liver, and the blood loss was determined by measuring the mass increase in the paper.

### 4.9. The Wound-Healing Analysis In Vivo

To assess wound-healing in vivo, the mice were randomly divided into three groups: a control group; the HG group; and the COT group. To establish a dorsal wound mouse model, mice with depilated backs were first anesthetized, and then full-thickness skin lesions measuring 10 mm in diameter were generated on their backs. The wound sites were then immediately covered with either COT hydrogel or HG (mice in the control group received no treatment). The hydrogels were refreshed daily, and the wound sizes were recorded on days 0, 1, 3, 5, 7, 9, and 13. Wound healing rates were calculated as follows:

(7) $$\mathrm{Healing}\;\mathrm{rate}\;(\%)=\frac{\left(S_{0}-S_{t}\right)}{s_{0}}\times 100\%$$

where S_0_ represents the original size of each wound site and S_t_ represents the area of each wound site on the day of recording.

After completion of the experiment, the skin and subcutaneous tissue at the wound healing site were excised for fixation, dehydration, clearing, and paraffin embedding. The tissue sections were subsequently stained with hematoxylin and eosin (H&E) and Masson’s trichrome stain, and histopathological changes were meticulously examined.

### 4.10. Statistical Analysis

All experimental data are expressed as mean ± standard deviation (SD). In all cases, one-way analysis of variance was used to evaluate the significance of differences in the mean (* *p* < 0.05, ** *p* < 0.01, *** *p* < 0.001).

## Figures and Tables

**Table 1:** The gelation time of COT-2 hydrogels (mean ± SD, *n* = 3).

Hydrogel	Ratio (CAF:OT, *v*/*v*)	Gel Time (s)
COT-2-0.5	1:2	>180
COT-2-1	1:1	>180
COT-2-2	2:1	60 ± 5
COT-2-3	3:1	20 ± 3
COT-2-4	4:1	36 ± 3
COT-2-8	8:1	>180

**Table 2:** The gelation time of COT-3 hydrogels (mean ± SD, *n* = 3).

Hydrogel	Ratio (CAF:OT, *v*/*v*)	Gel Time (s)
COT-3-0.5	1:2	>180
COT-3-1	1:1	45 ± 8
COT-3-2	2:1	13 ± 2
COT-3-3	3:1	15 ± 3
COT-3-4	4:1	70 ± 7
COT-3-8	8:1	>180

**Table 3:** Primary Skin Irritation Index of mice after multiple application (mean ± SD, *n* = 10).

Primary Skin Irritation Index																	
	1 d	2 d	3 d	4 d	5 d	6 d	7 d	8 d	9 d	10 d	11 d	12 d	13 d	14 d	Final Application		
															24 h	48 h	72 h
Control	0	0	0	0	0	0	0	0	0	0	0	0	0	0	0	0	0
HG	0	0	0.7 ± 0.78	0.8 ± 0.74	1.0 ± 0.77	1.0 ± 0.77	1.0 ± 0.77	0.6 ± 0.69	0.3 ± 0.46	0	0	0	0	0	0	0	0
COT	0	0	0	0	0	0	0	0	0	0	0	0	0	0	0	0	0

**Table 4:** The allergenicity scores and sensitization rates (*n* = 10).

	Total Skin Score				Average Skin Score				Sensitization Rate			
	1 h	24 h	48 h	72 h	1 h	24 h	48 h	72 h	1 h	24 h	48 h	72 h
Control	0	0	0	0	0	0	0	0	0	0	0	0
CNDNB	15	15	15	15	1.5	1.5	1.5	1.5	100	100	100	100
HG	0	0	0	0	0	0	0	0	0	0	0	0
COT	0	0	0	0	0	0	0	0	0	0	0	0

**Table 5:** The clotting time and BCI of hydrogels (mean ± SD, *n* = 6).

Groups	Clotting Time (s)	BCI (%)
Control	286.67 ± 17.51	None
HG	188.33 ± 14.72 ***	11.52 ± 2.84
COT	195.00 ± 18.71 ***	12.23 ± 3.49

Significance: *** *p* < 0.001 vs. the Control group.

**Table 6:** Four different concentration levels of CMCS, SA and Fuc.

Levels	Polysaccharides		
	CMCS (mg/mL)	SA (mg/mL)	Fuc (μg/mL)
1	30	15	30
2	20	10	20
3	10	5	10
4	0	0	0

**Table 7:** Orthogonal experimental design table.

Experiment Number	CMCS	SA	Fuc
1	1	1	1
2	1	2	2
3	1	3	3
4	1	4	4
5	2	1	2
6	2	2	1
7	2	3	4
8	2	4	3
9	3	1	3
10	3	2	4
11	3	3	1
12	3	4	2
13	4	1	4
14	4	2	3
15	4	3	2
16	4	4	1

**Table 8:** Grading scale for quantifying the degree of skin irritation.

Skin Reaction	Score
Erythema and eschar formation	
No erythema	0
Very slight erythema (barely perceptible)	1
Well-defined erythema	2
Moderate to severe erythema	3
Severe erythema (best redness) to slight eschar formation (injuries in depth)	4
Oedema formation	
No oedema	0
Very slight oedema (barely perceptible)	1
Slight oedema (edge of area well defined by definite raising)	2
Moderate oedema (raised approximately 1 mm)	3
Severe oedema (raised more than 1 mm and extending beyond the area of exposure)	4
Total possible score for irritation	8

**Table 9:** Skin irritation response scoring criteria.

Mean Score	Response Category
0–0.4	Negligible
0.5–1.9	Slightly
2–4.9	Moderate
5–8	Severe

**Table 10:** Magnusson and Kligman scales.

Topical Reaction	Score
Invisible Changes	0
Mild erythema	1
Moderate erythema	2
Severe erythema and edema	3

## Data Availability

The original contributions presented in this study are included in the article. Further inquiries can be directed to the corresponding authors.

## Abbreviations

The following abbreviations are used in this manuscript:

CMCS	Carboxymethyl chitosan
SA	Sodium alginate
Fuc	Fucoidan
OS	Oxidized starch
FTIR	Fourier Transform Infrared Spectroscopy
TA	Tannic acid
SEM	Scanning electron microscopy
BCI	Blood clotting index
DMEM	Dulbecco’s Modified Eagle’s Medium
UV	Ultraviolet
OD	Optical density
HE	Hematoxylin and eosin
